# Visual masking deficits in schizophrenia: a view into the genetics of the disease through an endophenotype

**DOI:** 10.1038/s41398-022-02275-4

**Published:** 2022-12-31

**Authors:** Albulena Shaqiri, Flavia Hodel, Janir Ramos da Cruz, Maya Roinishvili, Eka Chkonia, Andreas Brand, Jacques Fellay, Michael H. Herzog

**Affiliations:** 1 Laboratory of Psychophysics, Brain Mind Institute, EPFL, Lausanne, Switzerland; 2 Global Health Institute, School of Life Sciences, EPFL, Lausanne, Switzerland; 3 Swiss Institute of Bioinformatics, EPFL, Lausanne, Switzerland; 4 Wyss Center for Bio and Neuroengineering, Geneva, Switzerland; 5 Laboratory of Vision Physiology, Ivane Beritashvili Center of Experimental Biomedicine, Tbilisi, Georgia; 6 Institute of Cognitive Neurosciences, Free University of Tbilisi, Tbilisi, Georgia; 7 Department of Psychiatry, Tbilisi State Medical University, Tbilisi, Georgia; 8 Precision Medicine Unit, University Hospital and University of Lausanne, Lausanne, Switzerland

**Keywords:** Schizophrenia, Clinical genetics

## Abstract

Schizophrenia is a severe psychiatric disorder determined by a complex mixture of genetic and environmental factors. To better understand the contributions of human genetic variations to schizophrenia, we performed a genome-wide association study (GWAS) of a highly sensitive endophenotype. In this visual masking endophenotype, two vertical bars, slightly shifted in the horizontal direction, are briefly presented (vernier offset). Participants are asked to indicate the offset direction of the bars (either left or right). The bars are followed by a grating mask, which makes the task both spatially and temporally challenging. The inter-stimulus interval (ISI) between the vernier and the mask was determined in 206 patients with schizophrenia, 109 first-order relatives, and 143 controls. Usually, in GWAS studies, patients are compared to controls (i.e., a binary task) without considering the large differences in performance between patients and controls, as it occurs in many paradigms. The masking task allows for a particularly powerful analysis because the differences in ISI within the patient population are large. We genotyped all participants and searched for associations between human polymorphisms and the masking endophenotype using a linear mixed model. We did not identify any genome-wide significant associations (*p* < 5 × 10^−8^), indicating that common variants with strong effects are unlikely to contribute to the large inter-group differences in visual masking. However, we found significant differences in polygenetic risk scores (PRS) between patients and controls, and relatives and controls.

## Introduction

Schizophrenia is a heterogeneous psychiatric disorder strongly influenced by genetic factors. For example, there is a 30–50% risk that a monozygotic twin of a patient with schizophrenia will also develop the disease [1–3]. In addition, de novo mutations contribute strongly to the risk of developing schizophrenia [4].

Studies on the genetic basis of schizophrenia have evolved significantly from candidate gene strategies (for a review, see [5, 6]) to genome-wide association studies (GWAS, for reviews, see [7, 8]). Since 2005, GWAS have allowed unbiased interrogation of genome-wide variations and this has led to the identification of robust associations. The Psychiatric Genomics Consortium (PGC), for example, published a study in 2013 that identified 22 loci significantly associated with schizophrenia [9]. In 2014, the PGC genotyped more than 36,900 patients and identified 108 genome-wide significant loci, covering more than 600 genes [8, 10]. Since one genomic region (or loci) can be linked to multiple genes, the identification of specific genes and single-nucleotide polymorphisms (SNPs) involved in schizophrenia remains a very challenging task. Most of GWAS studies use a *binary* case-control design, in which a participant is considered as patient or healthy.

We suggest that endophenotypes could provide valuable additional information in genetic research of schizophrenia. Endophenotypes are measurable physiological or behavioral manifestations of disease-related genetic factors [11, 12]. Greenwood and colleagues [13], for example, tested for an association between a GWAS and 11 endophenotypes. Seven regions were significantly associated with endophenotypes after correction for multiple testing, such as the Continuous performance task (CPT) or the anti-saccade task.

We, here, used a visual backward masking endophenotype. A vernier, consisting of two bars, is presented, and the lower bar is offset either to the left or to the right. The vernier is followed by a mask and participants indicate the direction of the shift after the mask is presented. This task has a very high sensitivity (87%, *p* < 0.0001) and specificity (89%, *p* < 0.0005) for differentiating patients from controls [14]. The performance of relatives of the patients is between the one of the patients and controls, which is a first criterion for an endophenotype [11, 14, 15]. We also found that performance falls along a continuum based on the severity of the diagnosis: healthy students with a high schizotypy score perform worse compared to those with a low score [16]. Yet, students with high schizotypy perform much better than patients [17]. Performance deficits are also present in adolescents with psychosis, showing that the deficits are present in the early stages of the disease [18]. Masking deficits do not change over the course of one year in adult patients, meaning that this endophenotype is stable over time [14]. Finally, patients with functional psychosis, such as bipolar patients and schizoaffective patients, are deficient in the visual backward masking task, unlike depressive patients or abstinent alcoholics [14, 19]. All of the above meet the conditions for visual backward masking to be considered an endophenotype of schizophrenia [11]. Finally, in electroencephalography, the behavioral deficits are reflected by a smaller elicited N1 amplitude at around 200 ms after stimulus onset in patients compared to controls [20]. Similar differences between patients and controls were also found in individuals with a first episode of psychosis [21].

Here, we tested more than 450 participants with the visual backward masking endophenotype and CPT using GWAS. Our sample size is small compared to most contemporary GWAS comparing patients and healthy controls in a binary fashion. In contrast, our study investigates the impact of genetic factors on masking performance (endophenotype), which is, mathematically speaking, a real number. Our hypothesis was that the sensitivity and specificity of the endophenotype could allow of a much larger effect size on the alleles compared to the effect size in schizophrenia case-control studies, and thus, allow us to use a smaller sample size. Lastly, we were also interested in investigating the influence of multiple genetic markers on the three groups (patients with schizophrenia, unaffected relatives, and controls) and computed polygenic risk score (PRS) based on the results of an independent case-control GWAS on schizophrenia [10, 22].

## Methods

### Participants

214 patients with schizophrenia, 113 first-degree relatives of the patients, and 148 healthy controls participated in the study. Behavioral data of more than 150 participants were published previously [14, 15, 17, 19, 21, 23, 24]. The present analysis contains all data that were available in 2018 in our database. We excluded all participants with neurological disorders, traumatic brain injury or with a present history of drug or alcohol abuse. Eight patients, 4 relatives, and 5 controls were removed because they did not pass the genotyping quality control or did not have data on all the covariates (see the sections “Continuous performance test (CPT)” and “Single-nucleotide polymorphism (SNP) genotyping and quality control (QC)” for details on the quality control criteria). Therefore, we included 206 patients, 109 relatives, and 143 controls in the final analysis. Groups are described in Table 1. Patients were recruited from the Tbilisi Mental Health Center, Georgia. Patients were diagnosed according to DSM-IV by means of an interview based on the SCID, information of the staff, and the study of the records. Psychopathology of patients with schizophrenia was assessed by an experienced psychiatrist (EC) by Scales for the Assessment of Negative Symptoms and Scales for the Assessment of Positive Symptoms (SANS, SAPS [25, 26]). Relatives were asked to participate after the patients consented. All relatives and controls were free from psychiatric axis I disorders, but were not tested for axis II or schizotypic features. The relatives of patients with schizophrenia were 108 siblings and 5 parents, with some families contributing with more than one relative. One pair of monozygotic twins with schizophrenia was part of the study. Sixty-one patients with schizophrenia contributed relatives. Healthy controls were recruited from the general population. Ethics approval was obtained from the Georgian National Council on Bioethics. Participants signed informed consent and were informed that they could quit the experiments at any time.

**Table 1:** Participants’ characteristics.

	Schizophrenia patients (*N* = 206)		Relatives (*N* = 109)		Controls (*N* = 143)	
	Mean	Sd	Mean	Sd	Mean	Sd
Sex (F/M)	51/155	–	58/51	–	58/85	–
Age (yrs)	35.62	8.60	33.77	10.10	33.98	8.36
Age range (yrs)	16–55	–	16–60	–	17–55	–
Education (yrs)	13.15	2.85	14.25	3.73	14.97	2.86
Illness duration (yrs)	10.63	8.45	–	–	–	–
SANS	10.67	5.35	–	–	–	–
SAPS	9.31	3.36	–	–	–	–
CPZ	607.58	400.41	–	–	–	–
Visual acuity (both eyes)	1.41	0.39	1.57	0.36	1.53	0.41

The age, age range, education, and illness duration are reported in years. SANS refers to the Scale for the Assessment of Negative Symptoms; SAPS refers to the Scale for the Assessment of Positive Symptoms, CPZ refers to Chlorpromazine equivalents. Visual acuity is assessed for both eyes open.

### Visual backward masking task

**Fig. 1:**
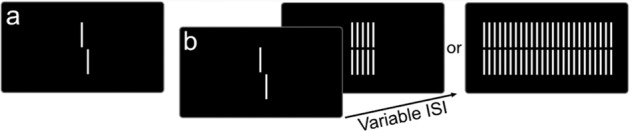
Stimuli. **a** Vernier stimuli consisting of two vertical bars offset to the right (or left not presented). **b** Visual backward masking with the grating consisting of either 5 or 25 elements.

Participants were seated at 3.5 m from the computer screen in a dimly illuminated room. The stimuli were white (100 cd/m^2^) on a black background. In a first step, we presented vernier stimuli consisting of two vertical bars of 10’ (arc min) of length, which were offset in the horizontal direction. Participants indicated via button press the offset direction of the lower bar compared to the upper bar (left or right). The offset direction was chosen randomly. Errors were indicated by an auditory signal. Participants performed 80 trials. For each observer, we determined the individual vernier duration (VD, in ms) to reach 75% correct responses using a staircase procedure (for details, see [27]). Observers with vernier durations longer than 100 ms were excluded at this stage to ensure that all observers attended to the stimuli (Fig. 1).

Once the individual vernier duration was established (20 ms is the minimal duration), the vernier was followed in the second step by a variable inter-stimulus interval (ISI), i.e., a blank screen, and then a grating for 300 ms. We varied the ISI adaptively using a staircase procedure [28]. In a separate session, we used two gratings consisting of either 5 or 25 verniers without offset of the same length as the target vernier. The horizontal distance between grating elements was about 3.33′. The outcome measure was the stimulus onset asynchrony (SOA = Individual vernier duration + ISI, in ms). The starting value of the SOA was 200 ms and then it either increased or decreased in order to find the individual threshold for each participant. Participants performed 80 trials. Each participant performed the test twice. First and second testing results were averaged and submitted to statistical analysis. We compared the performance across subjects with a 2-way repeated measures (rm)-ANOVA of the factors Group (patients, relatives, and controls) and Condition (five- and 25-element grating) with sex, age, education, and visual acuity as covariates.

The five-element grating (SOA5) leads to stronger masking than the 25-element grating (SOA25) even though the five-element grating is contained in the 25-element grating [29]. This difference in masking strength indicates that a substantial part of the masking power is not of retinal origin, because retinal processing is mainly determined by the sheer amount of light, e.g., the number of grating elements presented. We first established the individual vernier duration to have all participants perform at the same level. We used this procedure to make sure that potential deficits can be attributed to backward masking and were not caused by other elements.

### Freiburg visual acuity test (=FrACT)

This test has been developed and described in detail by Bach [30] and has been validated in various studies [31, 32]. FrACT is a computerized visual acuity (=VA) test capable of presenting large Landolt-C optotypes with randomized gap orientations on a computer monitor. In this study, the optotypes were presented on the computer screen and participants had to indicate the direction of the opening. There were four possible answers (‘up’, ‘down’, ‘left’, ‘right’), and participants were instructed to verbalize their responses, while the experimenter operated the input device. The size of each optotype presented always targets with the currently most probable VA threshold, calculated on the basis of all previous responses following a best-PEST algorithm [33].

### Continuous performance test (CPT)

Participants were tested with the degraded Continuous Performance Test CPT-DS (240 digits, 10% targets, degradation 40%) during 4 min. Observers had to detect the pair “1–9”, which means that they had to press a button when the digit 9 was presented just after the digit 1. The digits were presented randomly with a rate of one per second with a presentation time of 50 ms (for details, see [23]). We conducted also a GWAS for participants’ performance on the CPT. First, as the data were not normally distributed, we had to transform them using the box-cox method. Although the distribution was still not exactly normally distributed after transformation, this was the best fit. A one-way ANCOVA was conducted to determine the effect of Group (patients, relatives, and controls) on CPT performance controlling for sex, age, education, and FrACT.

### Single-nucleotide polymorphism (SNP) genotyping and quality control (QC)

All participants were genotyped using the Infinium Global Screening Array-24 v1.0 + Multi Disease (GSA + MD) array designed to human genome build 37. Genotyping of 700,078 single-nucleotide polymorphisms (SNPs) was performed at the iGE3 Genomics Platform of the University of Geneva (http://www.ige3.unige.ch/genomics-platform.php).

The quality control procedure described by Anderson and colleagues [34] was applied after removal of SNPs with missing or duplicated rsIDs. To confirm participants’ relationships and identify duplicate samples, the degree of shared ancestry between pairs of individuals (identity-by-state, IBS) was computed. We next identified samples with discordant sex information by computing the mean homozygosity rate across X-chromosome markers. Males should have an X-chromosome homozygosity estimate above 0.8 and females should have a value lower than 0.2. Individuals with a non-European ancestry were identified by merging data from 11 HapMap [35] version III populations (ASW, CEU, CHB, CHD, GIH, JPT, LWK, MXL, MKK, TSI, YRI) and conducting principal component analysis (PCA) on the merged individuals. Lastly, variants were excluded if they had a high missing data rate (>1%) in a large proportion of the subjects or if they deviated significantly from Hardy-Weinberg equilibrium (HWE, *P* < 10^−7^).

### Imputation of data for non-genotyped SNPs and quality control (QC)

To statistically estimate the haplotypes, individuals were first phased the genotypes using EAGLE2 [36] v2.0.5 software and the Haplotype Reference Consortium (HRC) [37] r1.1 reference panel. Then, to increase the resolution of the genetic association study, genotype imputation was used to infer missing genotypes from haplotype panels. Using Positional Burrows-Wheeler Transform (PBWT) and the HRC r1.1 reference panel, genotype data were imputed to more than 39 million SNPs on Sanger Imputation Server (https://imputation.sanger.ac.uk). Finally, to filter out poorly imputed genotypes, SNPs with imputation information score (INFO) < 0.8, minor allele frequency (MAF) < 5% or significant deviation from Hardy-Weinberg equilibrium (HWE, *p* < 10^−07^) were excluded.

### Genome-wide association study

Single-marker GWAS was conducted in GCTA [38] using a mixed linear model association analysis with the implemented leave-one-chromosome-out (LOCO) function. This SNP-by-SNP association testing, allowed to obtain a *p*-value computed for each SNP via linear regression, controlling for population stratification by considering the genetic relationship matrix (GRM) defined by all autosomal chromosomes except the chromosome on which the tested SNP is located. In other words, it prevents deflation due to double counting of the candidate SNP as both a fixed effect and a random effect in the mixed model. The model was adjusted for sex, age, education, FrACT, CPT and the top 3 principal components (PCs) of ancestry. Some individuals were excluded at this stage due to missing covariate values and GWAS was thus performed on 458 individuals: 206 patients with schizophrenia, 109 first-order relatives, and 143 controls. In the CPT GWAS, we adjusted the model for sex, age, education, FrACT, the visual backward masking task, and the top 3 principal components (PCs) of ancestry.

### Polygenic risk score

We constructed polygenic risk scores (PRS) for each individual in the study using the PRSice v2.2.7 software [39] to determine the degree of genetic risk an individual has for developing schizophrenia. The PRS were calculated by summing the number of risk alleles that an individual possesses, multiplied by the trait-specific weight (the β from the regression) as reported by the discovery data set. The calculations were based on common SNP risk effects derived from summary statistics from the meta-analysis of the CLOZUK [22] and the Psychiatric Genomics Consortium (PGC) data sets, and adjusted for sex, age, education, visual acuity, CPT degraded, and top 10 PCs of ancestry. Prior to the further analyses, the PRS were standardized to mean zero and standard deviation of one. An analysis of variance (ANOVA) test with Tukey’s post hoc tests was then used to compare mean PRS scores across cases, relatives, and controls. Nagelkerke’s R^2^ was used to compute the percentage of variance explained in SOA25 by the full model or covariates only.

We also used Pearson product-moment correlation to evaluate the correlation between the PRS scores of patients with schizophrenia, relatives, and controls with their performance on VBM and CPT.

### Machine learning to predict schizophrenia diagnosis

To assess the potential contribution of the SOA25 results and the previously calculated PRS results to predict schizophrenia, data excluding relatives were randomly divided with 80% (*N* = 284) used for training and 20% (*N* = 70) for validation. Data processing and machine learning were developed in R (version 4.1.1). Predictors were scaled to range between 0 and 1. Five common classifiers were chosen and their implementations were used via their interfacing with the open-source R *caret* package. The selection included classifiers commonly used in health data analysis and more advanced classifiers, including Multivariate adaptive regression splines (MARS), random forest (RF), support vector machine (SVM), eXtreme Gradient Boosting (XGBDART), and adaptive boosting (ADABOOST). All classifiers were run on each training data set using 3 separate 10-fold cross-validation schemes. To evaluate the prediction and accuracy of the different machine learning models, we calculated sensitivity and specificity using the testing data set, and compared the areas under the receiver-operating characteristic (ROC) curve.

## Results

### Behavioral results

#### Visual backward masking

**Fig. 2:**
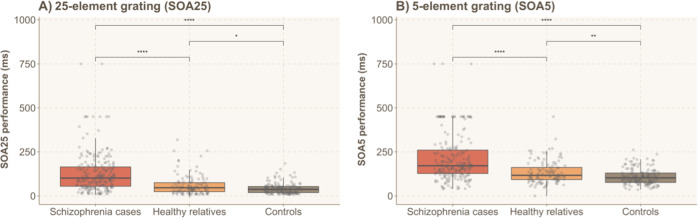
Threshold SOAs for the 25 and 5 elements grating (SOA 25 and SOA 5, respectively). Patients had longer SOAs compared to their relatives and controls for both the task **A** with the 25 and **B** with 5 gratings. Performance of relatives lies between the one of controls and patients. Asterisks indicate statistically significant differences: *P < 0.05*,* **P ≤ 0.01, ***P ≤ 0.001*,* ****P ≤ 0.0001, n.s. not significant.

Since the data were not normally distributed, we log transformed them for statistical analysis. Differences in SOA between groups are shown in Fig. 2. A two-way rm-ANOVA showed significant effects of Group (F(2, 451) = 68.509, *p* = 1.045e−26, *η*^2^ = 0.177) and Condition (F(1, 451) = 8.151, *p* = 0.005, *η*^2^ = 0.003) as well as a significant Group × Condition interaction (F(2, 451) = 20.838, *p* = 2.210e−9, *η*^2^ = 0.014). The interaction indicates that Group differences depended on the Condition. For the 25 elements grating, simple main effects of Group indicated that there was a significant group difference (F(2, 451) = 61.539, *p* = 2.338e−24, *η*^2^ = 0.200) with patients with schizophrenia having the longest SOA (129.45, ±114.95) compared to their relatives (60.61, ±57.26) and controls (41.95, ±30.54). A post hoc analysis, Bonferroni-Holm corrected for multiple comparisons, revealed that patients were different from the two other groups (*p* = 5.901e−23 for controls, Cohen’s d = 1.184 and *p* = 2.876e−13 for relatives, d = 0.865) and relatives were different from controls (*p* = 0.045, d = 0.262). An identical analysis revealed similar results for the 5 elements grating (F(2, 451) = 50.240, *p* = 2.00e−20, *η*^2^ = 0.172). Patients had longer SOAs (212.03, ±123.77) compared to their relatives (131.96, ±61.69) and controls (108.80, ±44.40). The three groups were significantly different from each other (*p* = 6.359e−20 for patients and controls, d = 1.038, and *p* = 7.692e−10 for patients and relatives, d = 0.720 and *p* = 0.014 and d = 0.473 for relatives and controls).

We also tested for gender differences within groups. In the patient group, males and females did not perform differently with the 25 or 5 elements gratings (all *p* > 0.27). Males and females of the relatives group were different for the 5 elements gratings (F(1, 107) = 8.66, *p* = 0.004), with females having longer SOAs than males. Finally, in the control group, males had a shorter SOA than females for the 25 or 5 elements gratings (all *p* values < 0.0001).

Additionally, we tested a similar model using sex as a factor instead of a covariate. In the backward masking task, this three-way rm-ANOVA showed a significant effect Group (F(2, 449) = 55.370, *p* = 3.208e−22, *η*^2^ = 0.148) and Condition (F(1, 449) = 16.724, *p* = 5.125e−5, *η*^2^ = 0.006) as well as a significant Group × Condition interaction (F(2, 449) = 16.119, *p* = 1.726e−7, *η*^2^ = 0.012). However, there was no significant Group × Condition × Sex interaction (F(2, 449) = 1.565, *p* = 0.210, *η*^2^ = 0.001). For the 25 elements gratings, a two-way ANCOVA indicated a significant main effect of Group (F(2, 449) = 49.291, *p* = 4.440e−20, *η*^2^ = 0.167) and Sex (F(1, 449) = 11.471, *p* = 7.692e−4, *η*^2^ = 0.019) but no significant Group × Sex interaction (F(2, 449) = 1.866, *p* = 0.156, *η*^2^ = 0.006). Similarly, for the 5 elements gratings, a two-way ANCOVA indicated a significant main effect of Group (F(2, 449) = 41.254, *p* = 3.569e−17, *η*^2^ = 0.149) and Sex (F(1, 449) = 11.467, *p* = 7.708e−4, *η*^2^ = 0.020) but no significant Group × Sex interaction (F(2, 449) = 0.705, *p* = 0.495, *η*^2^ = 0.002). All in all, these results suggested that the group differences do not depend on the sex.

#### CPT

**Fig. 3:**
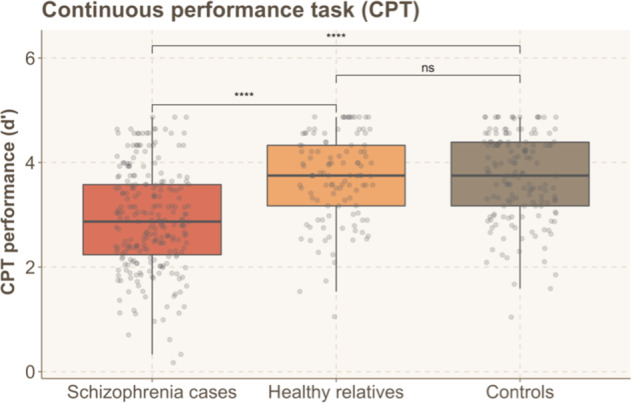
Performance on CPT for the 3 groups. Patients performed significantly differently from their relatives and controls; relatives and controls performed similarly. Asterisks indicate statistically significant differences: *P < 0.05, **P ≤ 0.01, ***P ≤ 0.001, ****P ≤ 0.0001, n.s. not significant.

For the continuous performance task, a one-way ANCOVA revealed significant effect of group (F(2, 451) = 31.449, *p* = 1.637e−13, *η*^2^ = 0.116). Post hoc analysis, Bonferroni-Holm corrected for multiple comparisons, showed that patients with schizophrenia performed worse (2.99 ± 0.95) than their relatives (3.73 ± 0.79; *p* = 1.392e−10, d = 0.809) and controls (3.71 ± 0.83; *p* = 1.506e−10, d = 0.731). Performance of relatives and controls were at a similar level (*p* = 0.541, d = 0.083). Performance on the CPT task is shown in Fig. 3. As for gender differences within groups, only the control group was different between males and females (F(1, 141) = 8.1, *p* = 0.005), with females performing worse than males.

For the CPT, we additionally tested a model using sex as a factor instead of a covariate. This two-way ANCOVA revealed a significant effect Group (F(2, 449) = 24.245, *p* = 1.003e−10, *η*^2^ = 0.092) and Sex (F(1, 449) = 5.154, *p* = 0.024, *η*^2^ = 0.010) but no significant Group × Sex interaction (F(2, 449) = 1.937, *p* = 0.145, *η*^2^ = 0.007). These results suggested that the group differences do not depend on the sex.

### Genome-wide associations study (GWAS) results

We genotyped all participants and searched for associations between human polymorphisms and masking SOA. Following the quality control steps (see “Methods”), one duplicate sample was removed after computing the degree of shared ancestry between pairs of individuals, while relatives and the pair of monozygotic twins were kept for the analyses. Two more individuals were excluded because of discrepancies between sex of the individuals recorded in the data set and their sex based on the mean X chromosome homozygosis rate (Supplementary Figure 1). We also excluded one additional individual due to elevated rate of genotype missingness (>5%). Supplementary Figure 2 shows the principal component analysis (PCA) obtained to assess the level of stratification in the study population. With this procedure, no reported data for any individual was excluded since no population outliers were observed in the sample distribution. Lastly, 13 individuals were removed because of missing data for at least one covariate. On the SNP side, we excluded those with high missingness (>1% of study participants) or with significant deviation from Hardy-Weinberg equilibrium (HWE, *p* < 10^−7^), resulting in approximately 620,000 SNPs and 458 individuals (206 cases, 109 relatives and 143 controls) that passed pre-imputation QC steps.

Genotype data were then imputed to more than 39 million SNPs (see “Methods”). A final QC was performed considering individual SNP imputation accuracy, MAF, and HWE, reducing the number of SNPs for the GWAS to ~3.5 million.

**Fig. 4:**
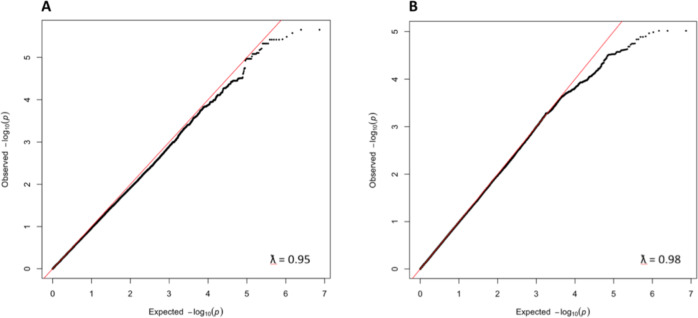
Quantile–quantile (Q–Q) plots from the GWAS study. Q–Q plots of SNPs for association to SOA25 (**A**) and SOA5 (**B**) in the data set consisting of 458 individuals. On the y-axis, the observed *p*-values (black dots) are plotted against the expected *p*-values under the null distribution. The straight red lines indicate the distribution of SNPs under the null distribution. Genomic inflation factor lambda (λ) are indicated in the bottom right corner.

**Fig. 5:**
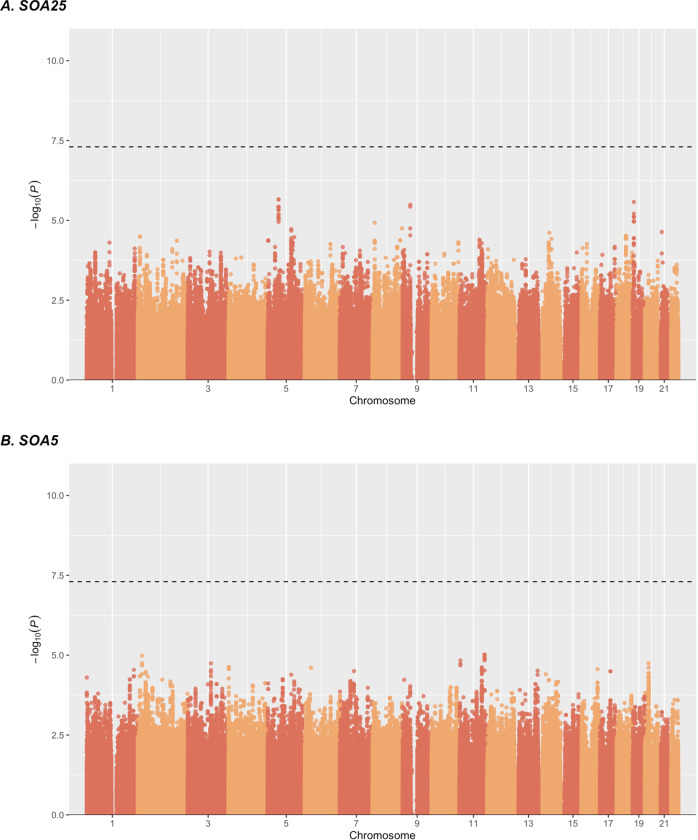
Manhattan plots. Manhattan plots showing the significance of association of all SNPs across chromosomes 1 to 22 in the analysis with SOA25 (**A**) and SOA5 (**B**) in 458 individuals. SNPs are plotted on the x-axis according to their position on each chromosome and associations with SOA25 and SOA5 are indicated on the y-axis (as −log10 *P* value). The dashed horizontal lines mark the genome-wide significance level of 5e^−08^.

We used GWAS to evaluate the contribution of common genetic variants across the entire genome to performance at the visual masking task. Sex, age, education, FrACT, CPT, and the top 3 PCs were included as covariates. The Q–Q plots in Fig. 4 show the observed distribution of the associations between genotyped SNPs and SOA25 (panel A) and SOA5 (panel B) values (Y-axes), compared to the association statistics expected under the null hypothesis of no association (X-axes), revealing whether SNPs deviate from the distribution under the null hypothesis. Genomic inflation factors λ of 0.95 and 0.98 for SOA25 and SOA5, respectively, indicate that the distributions of P-values are similar to chance expectation. The Manhattan plots of the GWAS (Fig. 5) summarize association findings by physical position of the SNPs. The top 25 findings for SOA25 and SOA5 are listed in Supplementary Table 1 and Supplementary Table 2, respectively. No SNP reached the genome-wide significance threshold of *p* = 5e^−8^.

As an additional analysis, we considered all the SNPs previously reported by the PGC (2014) to be associated with schizophrenia. Out of the 128 identified SNPs, 88 were present in our imputed and filtered data set, but none passed the corrected nominal significance threshold for association with SOA25 and SOA5.

**Fig. 6:**
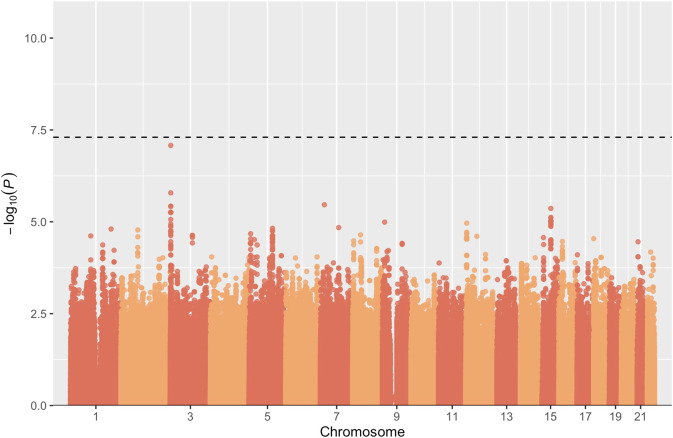
Manhattan plot showing the significance of association of all SNPs across chromosomes 1 to 22 in the analysis CPT in 458 individuals. SNPs are plotted on the x-axis according to their position on each chromosome and associations with CPT are indicated on the y-axis (as −log10 *P*). The dashed horizontal line marks the genome-wide significance level of 5e^−08^.

CPT GWAS results were similar to the VBM results. Sex, age, education, FrACT, VBM task, and the top 3 PCs were included in the linear model as covariates. The Manhattan plot of the GWAS for the CPT (Fig. 6) summarizes association findings by physical position of the SNPs. No SNP reached the genome-wide significance threshold of *p* = 5e^−08^.

### Polygenic risk score (PRS) results

PRS were calculated at thresholds that provide the best-fit PRS. A total of 2594 SNPs were included in the analysis of SOA25 at the best *P*-value threshold (*p* = 3.27e−05). The PRS followed a normal distribution in controls, healthy relatives and cases. We quantified the trait variance (R^2^) explained by the derived PRS across individuals and observed that the variance explained by the full model is 21%. The vast majority (18%), however, was attributed to the included covariates.

**Fig. 7:**
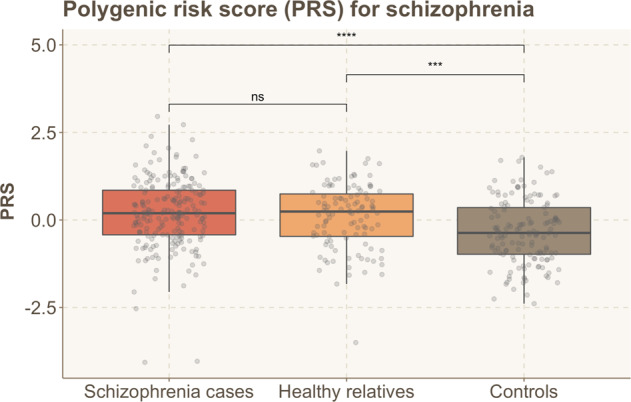
Boxplots of PRS for patients with schizophrenia, healthy relatives, and controls. Pairwise comparison *p*-values are indicated on top. Asterisks indicate statistically significant differences: *P < 0.05, **P ≤ 0.01, ***P ≤ 0.001, ****P ≤ 0.0001, n.s. not significant.

Using ANOVA, we found significant differences in PRS between the groups (*p* = 1.41e^−06^). In the advanced analysis (post hoc Tukey multiple comparisons of means), we found significant differences in mean PRS between patients with schizophrenia and controls (*p* = 1.23e^−06^), and between relatives and controls (*p* = 9.43e^−04^) but no statistically significant difference between patients and relatives (*p* = 0.69; Fig. 7).

PRS scores correlated with the performance on the VBM task (SOA25: R = 0.16, *p* = 4.42e^−04^; SOA5: R = 0.16, *p* = 7.57e^−04^). No significant association was found between the PRS scores and the CPT performance (R = −0.05, *p* = 0.30).

### Predictive modeling of schizophrenia from genomic data

We used five machine-learning algorithms to attempt to distinguish schizophrenia patients from controls using the previously calculated PRS and 25 element network results. After running the algorithms on the training data set, models were created and evaluated on the test data set. The best performance was achieved by eXtreme Gradient Boosting that reached a classification accuracy of 81.40%. Seventy individuals were correctly classified as patients and controls, compared to 16 misclassified individuals (Supplementary Figure 4).

## Discussion

**Fig. 8:**
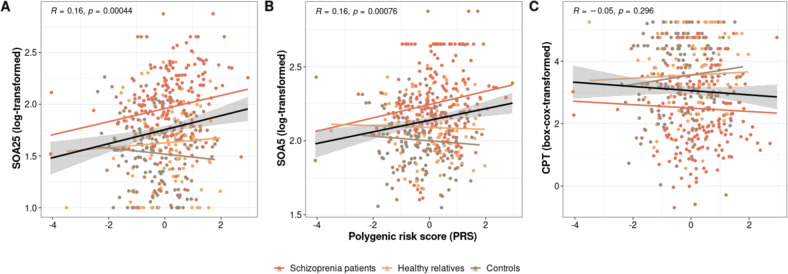
Polygenic risk score (PRS) versus 25 and 5 elements grating (SOA25 and SOA5), and continuous performance test (CPT). Weighted linear regressions using PRS as a predictor for **A** SOA25, **B** SOA5, and **C** CPT. Separate lines and points for each group are colored according to legend. The black line (with confidence interval) represents the overall line of best fit. Overall Pearson correlation coefficient (R) with significance (*p*) is presented in each box.

Schizophrenia is a heterogeneous disorder strongly influenced by genetic factors. Research on the genetics of schizophrenia has evolved very rapidly over the past decade. One of the most important studies published is the PGC study, which collected data from over 36,900 patients and identified 108 genome-wide significant loci [10]. Most of the significant loci showed very small effect sizes. In these studies, patients were compared to controls, i.e., a binary categorization, without considering the large differences between patients found in many paradigms, reflecting the heterogeneity of the disease in terms of psychopathology and cognition. For this reason, we hypothesized that the highly sensitive visual backward masking endophenotype may show potential associations between its large range of performance levels and human genetic variants. However, this hypothesis turned out to be wrong (Fig. 8).

Previous studies have assessed the association between various endophenotypes of schizophrenia and genetic factors. McDonald and colleagues [40] used white and gray matter as endophenotypes and associated structural points with genetic aspects in 25 patients and 36 of unaffected relatives. They reported that gray matter in the left temporal regions and in the bilateral fronto-striato-thalamic region was associated with genetic risks for schizophrenia. In a review, Gur et al. [41], reported a list of cognitive endophenotypes, such as attention or working memory, to be associated with genetics risks for schizophrenia. None of the studies took into account the large differences in performance between patients. For the visual backward masking endophenotype, we grouped all participants and then determined whether they belonged to the control group, the relative group, or the patient group, and then investigated whether specific genes were associated with performance on this task.

It has been reported that the nicotinic system may be deficient in patients with schizophrenia [42–44] and studies found a deletion of 15q13.3 (chromosomal location of CHRNA7) [45]. In a previous study, we investigated performance in the visual backward masking task in association with variants of the alpha 7 subunit of the cholinergic receptor (CHRNA7) [24]. We found that performance on the visual backward masking task was correlated with the SNP rs904952, which is a SNP in the nicotinic cholinergic receptor gene [24]. Unfortunately, we were unable to test for replication of this SNP in the present study, as rs904952 did not pass quality controls (see “Methods” section). We also compared the 128 independent associations of the PGC 2014 study with our results and found no significant association, which could be due to our small sample size.

The difficulty in identifying specific genes involved in schizophrenia may also be due to the fact that de novo mutations contribute strongly to the genetics of schizophrenia. Xu et al. [46], for example, found that de novo mutations were 8 times more frequent in patients with schizophrenia than in controls, which introduces new cases of schizophrenia into the population (for people who do not have the family genetic background making them susceptible to the disease).

Another reason might be that most of the genetic risk factors for schizophrenia also seem to be involved in other psychiatric disorders, which makes it difficult to isolate those that are only involved in schizophrenia. This is also the case for common and rare variants [47]. These results show that performance levels in backward masking cannot be explained by single genes but probably have a polygenic basis (for polygenic risk see [48]). However, we observed significant differences in PRS scores between cases and controls, and between healthy relatives and controls. Consistent with other studies, patients with schizophrenia have higher risk scores than controls, confirming that PRS has good potential to discriminate between schizophrenia cases and controls [49–52]. Similarly, unaffected relatives also have a higher PRS as compared to controls, supporting the hypothesis that healthy relatives are at high genetic risk given the heritability of schizophrenia and its familial aggregation patterns [49, 50]. Using the threshold of 3.27e^−05^, defined by PRSice-2, more than 2500 SNPs were included in the analysis, which is very large but not surprising, given the polygenicity of schizophrenia. We, therefore, confirmed using PRS that schizophrenia is the consequence of a large number of SNPs, each with a minor effect, which, in combination, create a vulnerability to schizophrenia in an individual. The individual minor effect of each SNP could also explain why we did not observe any genome-wide significant signal in the GWAS with this small sample size. However, we found that SNP scores were weakly correlated with VBM performance—but not with CPT—reinforcing the idea that VBM deficits are linked to an underlying genetic risk for schizophrenia, and thus constitute a potential endophenotype for the disorder [14]. Altogether, our results suggest that there is a continuum of genetic risk for schizophrenia and that PRS may be a promising tool to help clinicians diagnose individuals at high risk of developing schizophrenia, allowing for targeted prevention and effective early intervention.

We evaluated the performance of SOA25 and PRS combination in an attempt to accurately classify individuals in patients and controls. Our results implied that a machine learning algorithm trained with an endophenotype and genotyping data could fairly well discriminate patients with schizophrenia from healthy controls. These results could therefore complement other existing tools such as magnetic resonance imaging (MRI) data [53], multivariate voxel-based morphometry [54], or resting-state functional network connectivity [55] to classify individuals into cases and controls for an increasingly accurate diagnosis of the disease, which to date still lacks an objective biological diagnostic test.

Finally, we expected the polygenetic risk score to correlate with masking performance in both the patients’ and relatives’ groups. However, we only found a weak overall (all groups together) correlation between masking performance and polygenetic risk scores. This correlation was only significant for the patients’ group, when analyzing the groups individually. However, we do not think that these results suggest that masking performance is not related to polygenetic risk scores of schizophrenia, since we find a (weak) correlation between the two. We believe that there is an effect, but the effect is small. Moreover, in the relatives group, we speculate that there might be other effects (e.g., compensatory effects) that might affect masking performance. For example, in previous works, we found that the strong masking performance deficits in patients are accompanied with decreased ERP amplitudes compared to controls. However, da Cruz et al. [15] found that while performing a less challenging version of the visual backward masking used in the current paper, siblings of schizophrenia patients had increased ERP amplitudes compared to controls, while their performance were similar. Moreover, the ERP amplitudes in siblings were correlated with the performance, suggesting that siblings can partially compensate for the deficits by over-activating a network of brain regions.

Our study has limitations. First, the number of participants tested is rather limited for a GWAS, compared to other studies with thousands of participants [56]. However, we have the advantage of having a very sensitive endophenotype. Moreover, testing a very large number of participants with the latter would be almost impossible, if we want to keep other factors similar, such as geographical proximity of participants. Second, we could not test our hypothesis about the involvement of the cholinergic system in schizophrenia, as the SNPs we had identified in a previous study did not pass the quality control. Third, heritability, defined as the proportion of variation in the endophenotype explained by inherited genetic variants, could not be reliably calculated due to the relatively small sample size. Fourth, our PRS results could not be replicated by cross-validation due to sample size limitations. Finally, we used a very short version of the CPT (4 min), which does not allow for testing of sustained attention. We like to mention that, however, deficits of patients are present already in the first minutes of testing [23].

In conclusion, we used a highly sensitive endophenotype, visual backward masking, and another test, the CPT, which is considered as an endophenotype by some studies (for a review, see [23]) to test for an association between our tasks and millions of genetic variants. We did not identify any significant genome-wide association, indicating that common variants of single genes with large effects are unlikely to contribute to the large inter-individual differences observed in schizophrenia-related visual masking deficits.

## Supplementary information

Supplementary Material
