# Supplementary material for: Visual masking deficits in schizophrenia: a view into the genetics of the disease through an endophenotype
Source: Transl Psychiatry. 2022 Dec 31;12:529. doi: 10.1038/s41398-022-02275-4 (PMC9803632; doi:10.1038/s41398-022-02275-4)
Supplement: Supplementary file 1 — Supplementary Material [file 41398_2022_2275_MOESM1_ESM.docx]

**Supplementary material**


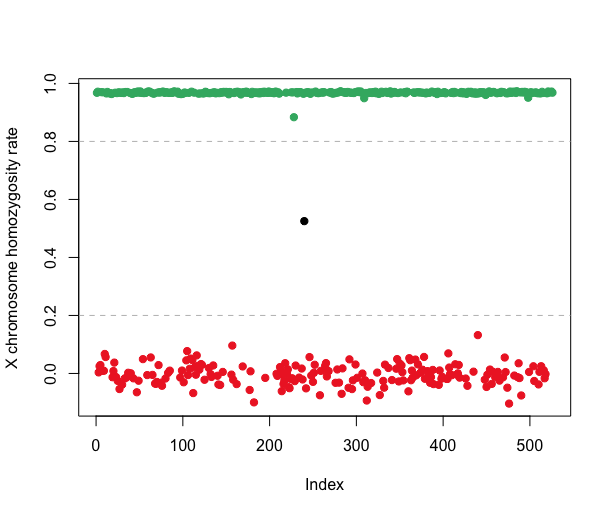


**Figure S1. X homozygosity rate per individual.** Men (green dots) have a homozygosity rate above 0.8 and women (red dots) have a homozygosity rate below 0.2. One individual (black dot) was identified as possible Klinefelter syndrome (XXY male).


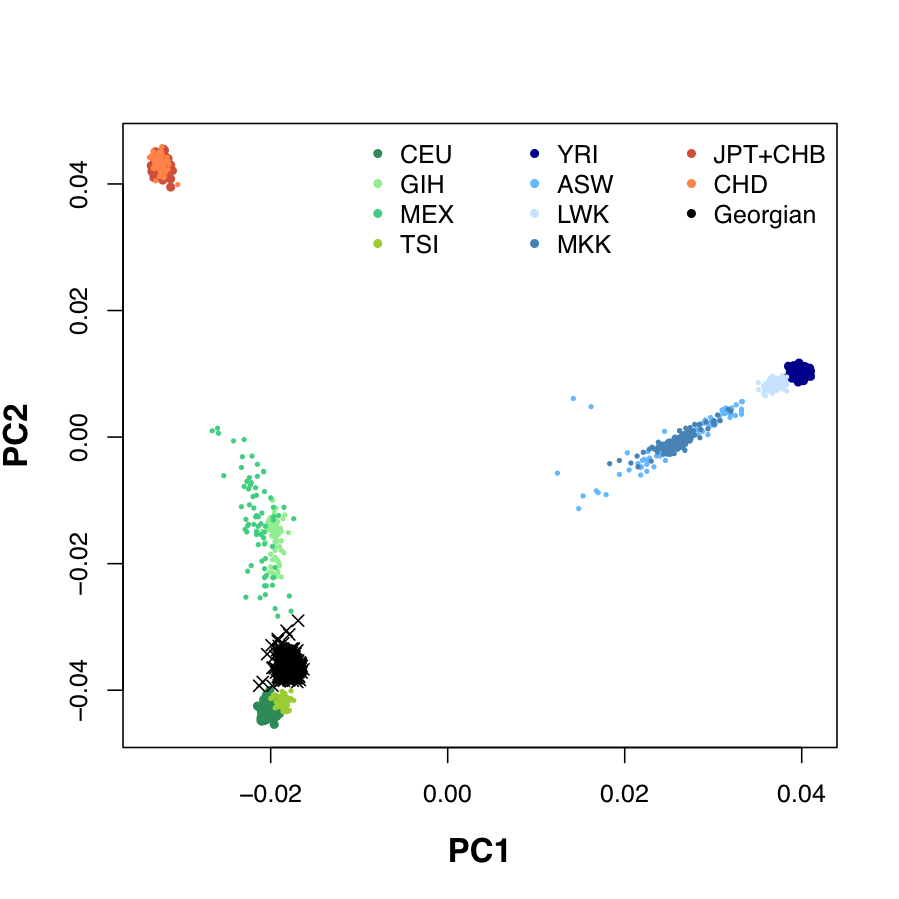


**Figure S2. Principal component analysis (PCA) plot of the Georgian study samples and HapMap3 samples.** ASW: African American, CEU: Utah Residents, CHB: Han Chinese, CHD: Chinese, GIH: Gujarati, JPT: Japanese, LWK: Luhya, MEX: Mexican American, MKK: Maasai, TSI: Toscani, YRI: Yoruba.

**Supplementary Table 1. Genome-wide associations study (GWAS) summary statistics for 25-element grating (SOA25).**

| **Chr** | **SNP** | **bp** | **A1** | **A2** | **Freq** | **b** | **se** | **p** |
| --- | --- | --- | --- | --- | --- | --- | --- | --- |
| 5 | rs372370 | 52903922 | A | G | 0.293668 | 0.117757 | 0.024882 | 2.22E-06 |
| 5 | rs435713 | 52903804 | A | C | 0.293668 | 0.117757 | 0.024882 | 2.22E-06 |
| 19 | rs113123495 | 5587540 | T | C | 0.0502183 | -0.253795 | 0.0540475 | 2.66E-06 |
| 9 | rs7045259 | 36886065 | C | T | 0.232533 | 0.123413 | 0.026515 | 3.25E-06 |
| 9 | rs9696279 | 36916871 | A | G | 0.234716 | 0.122621 | 0.0265049 | 3.72E-06 |
| 5 | rs10036570 | 52869005 | A | G | 0.293668 | 0.114433 | 0.0247554 | 3.79E-06 |
| 5 | rs13164833 | 52871414 | C | T | 0.293668 | 0.114433 | 0.0247554 | 3.79E-06 |
| 5 | rs256094 | 52972924 | A | G | 0.293668 | 0.114433 | 0.0247554 | 3.79E-06 |
| 5 | rs6864497 | 52882588 | C | T | 0.293668 | 0.114433 | 0.0247554 | 3.79E-06 |
| 5 | rs9292024 | 52869172 | T | A | 0.293668 | 0.114433 | 0.0247554 | 3.79E-06 |
| 5 | rs2636993 | 52929796 | A | G | 0.29476 | 0.113371 | 0.0247665 | 4.70E-06 |
| 5 | rs3103601 | 52891848 | G | A | 0.29476 | 0.113371 | 0.0247665 | 4.70E-06 |
| 5 | rs365578 | 52937809 | T | G | 0.29476 | 0.113371 | 0.0247665 | 4.70E-06 |
| 5 | rs409313 | 52899939 | T | A | 0.29476 | 0.113371 | 0.0247665 | 4.70E-06 |
| 5 | rs412559 | 52913935 | A | G | 0.29476 | 0.113371 | 0.0247665 | 4.70E-06 |
| 19 | rs113027608 | 5578403 | G | C | 0.0611354 | -0.224154 | 0.0495902 | 6.18E-06 |
| 5 | rs1490769 | 52954280 | A | G | 0.295852 | 0.111744 | 0.0247779 | 6.49E-06 |
| 5 | rs566111 | 53000047 | A | G | 0.293668 | 0.111469 | 0.0247554 | 6.71E-06 |
| 19 | rs10411903 | 5578099 | G | A | 0.069869 | -0.209424 | 0.046856 | 7.84E-06 |
| 19 | rs61705252 | 5579696 | A | C | 0.069869 | -0.209424 | 0.046856 | 7.84E-06 |
| 19 | rs7251910 | 5575246 | T | C | 0.069869 | -0.209424 | 0.046856 | 7.84E-06 |
| 5 | rs2219359 | 53003486 | C | T | 0.29476 | 0.110405 | 0.0247665 | 8.28E-06 |
| 5 | rs2607491 | 53003810 | C | T | 0.29476 | 0.110405 | 0.0247665 | 8.28E-06 |
| 5 | rs2637016 | 53004433 | C | T | 0.29476 | 0.110405 | 0.0247665 | 8.28E-06 |
| 5 | rs2637021 | 52998994 | G | T | 0.29476 | 0.110405 | 0.0247665 | 8.28E-06 |

Chr, chromosome; SNP, rsID; bp; base-pair position (build 37); A1, minor allele; A2, major allele; Freq, allele frequency of A1; b, effect size of A1; se, standard error of b; p, p-value.

The Genome Reference Consortium Human build 37 (GRCh37) is the reference genome used.

**Supplementary Table 2. Genome-wide associations study (GWAS) summary statistics for 5-element grating (SOA5).**

| **Chr** | **SNP** | **bp** | **A1** | **A2** | **Freq** | **b** | **se** | **p** |
| --- | --- | --- | --- | --- | --- | --- | --- | --- |
| 11 | rs73013971 | 122071924 | A | G | 0.34607 | -0.0686285 | 0.0155094 | 9.65E-06 |
| 11 | rs73013973 | 122071925 | T | G | 0.34607 | -0.0686285 | 0.0155094 | 9.65E-06 |
| 11 | rs73013975 | 122072103 | A | G | 0.34607 | -0.0686285 | 0.0155094 | 9.65E-06 |
| 2 | rs981396 | 20346651 | A | G | 0.407205 | -0.0632626 | 0.014344 | 1.03E-05 |
| 11 | rs61909866 | 122061369 | T | C | 0.362445 | -0.0658951 | 0.0149793 | 1.09E-05 |
| 11 | rs79357510 | 122031447 | C | G | 0.337336 | -0.0654675 | 0.0149944 | 1.26E-05 |
| 11 | rs17337634 | 122067773 | C | G | 0.348253 | -0.0673936 | 0.0154565 | 1.30E-05 |
| 11 | rs61909878 | 122068423 | C | T | 0.348253 | -0.0673936 | 0.0154565 | 1.30E-05 |
| 11 | rs61910054 | 122030746 | G | A | 0.335153 | -0.0648357 | 0.0149556 | 1.46E-05 |
| 11 | rs11032301 | 4472581 | C | G | 0.155022 | 0.0836668 | 0.0193037 | 1.46E-05 |
| 2 | rs17697993 | 20341993 | A | G | 0.409389 | -0.0614192 | 0.014315 | 1.78E-05 |
| 3 | rs1154275 | 112485222 | A | G | 0.473799 | 0.0630248 | 0.0146963 | 1.80E-05 |
| 20 | rs59832377 | 18571333 | C | T | 0.460699 | 0.0617454 | 0.0143986 | 1.80E-05 |
| 11 | rs11032304 | 4472880 | C | T | 0.156114 | 0.081921 | 0.019151 | 1.89E-05 |
| 11 | rs11032339 | 4482342 | G | A | 0.158297 | 0.0814252 | 0.0191281 | 2.07E-05 |
| 2 | rs6531213 | 20344011 | C | T | 0.407205 | -0.0610806 | 0.0143496 | 2.08E-05 |
| 4 | rs28522740 | 1569985 | A | G | 0.39083 | -0.0620043 | 0.01466 | 2.34E-05 |
| 4 | rs10003680 | 1588977 | T | C | 0.41048 | -0.0607757 | 0.0143857 | 2.39E-05 |
| 4 | rs6852987 | 1587323 | C | T | 0.41048 | -0.0607757 | 0.0143857 | 2.39E-05 |
| 11 | rs2553760 | 108753316 | A | G | 0.161572 | -0.0795548 | 0.018847 | 2.43E-05 |
| 11 | rs2553761 | 108754443 | A | G | 0.161572 | -0.0795548 | 0.018847 | 2.43E-05 |
| 20 | rs6136418 | 18560114 | A | G | 0.472707 | 0.0606522 | 0.0143792 | 2.46E-05 |
| 4 | rs10010688 | 1588848 | A | T | 0.411572 | -0.0607547 | 0.0144074 | 2.48E-05 |
| 6 | rs9258740 | 29827899 | T | C | 0.149563 | 0.0920321 | 0.0218247 | 2.48E-05 |
| 4 | rs57766696 | 1584640 | A | G | 0.415939 | -0.0606859 | 0.0144254 | 2.59E-05 |

Chr, chromosome; SNP, rsID; bp; base-pair position (build 37); A1, minor allele; A2, major allele; Freq, allele frequency of A1; b, effect size of A1; se, standard error of b; p, p-value.

The Genome Reference Consortium Human build 37 (GRCh37) is the reference genome used.


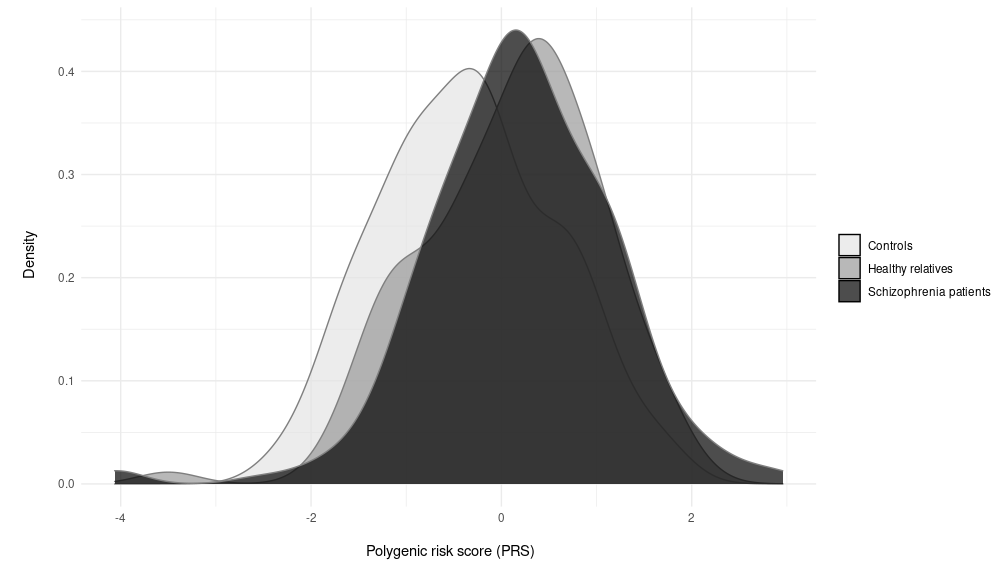


**Figure S3. Distribution of PRS scores in controls, healthy relatives and patients with schizophrenia.**


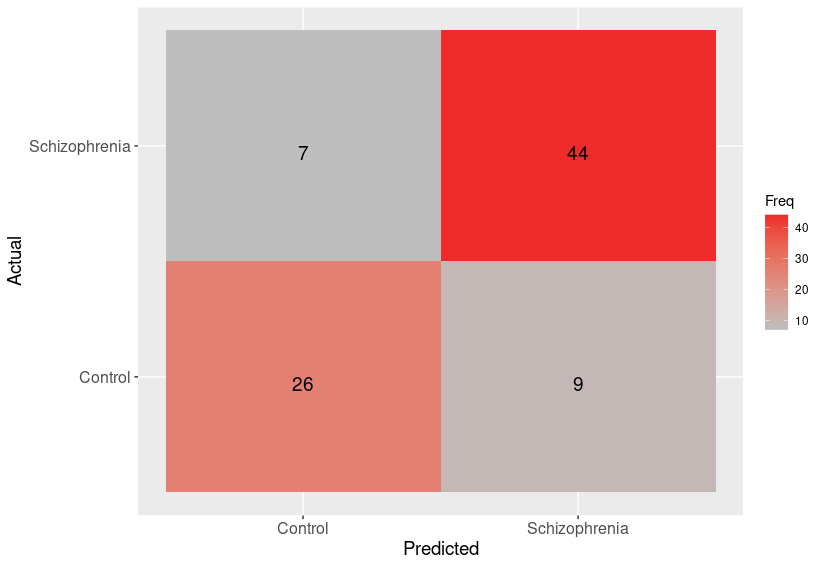


**Figure S4. Confusion matrix for Machine Learning classification of schizophrenia based on SOA25 and PRS for each participant in the test data set.** The rows represent the actual diagnosis (schizophrenia diagnosis vs. control) and the columns represent the predicted diagnosis. The number in each cell represents the number of participants for each scenario. Cases are colored by their frequency.
